# Farmers’ knowledge, perception, and use of phosphorus fertilization for cowpea production in Northern Guinea Savannah of Nigeria

**DOI:** 10.1016/j.heliyon.2020.e05207

**Published:** 2020-10-17

**Authors:** Saba B. Mohammed, Ishiyaku F. Mohammad, Tongoona B. Pangirayi, Gracen Vernon, Daniel K. Dzidzienyo, Muhammad L. Umar, Sulaiman Umar

**Affiliations:** a West Africa Centre for Crop Improvement, University of Ghana, Accra, Ghana; b Department of Plant Science, Faculty of Agriculture/Institute for Agricultural Research, Ahmadu Bello University, Zaria, Nigeria; c Department of Agricultural Extension and Rural Development, Faculty of Agriculture/Institute for Agricultural Research, Ahmadu Bello University, Zaria, Nigeria

**Keywords:** Adoption, Cowpea, Focus group discussion, Phosphorus-based fertilizers, Northern Nigeria, Root nodules, Agricultural economics, Agricultural policy, Agricultural soil science, Crop production, Field crops, Agricultural science, Plant biology

## Abstract

Cowpea (*Vigna unguiculata* L. Walp) is an important legume crop, especially in sub-Saharan Africa. Poor soil fertility is among the major abiotic factors that contribute to this crop's low yield. Phosphorus (P)-based fertilizers significantly increase cowpea yields but these fertilizers are not well adopted by smallholder cowpea farmers. To understand why, we surveyed 420 farmers across three major cowpea-producing states in Nigeria: first, we assessed the cowpea farmers' knowledge and perception of the need for fertilizers, especially P fertilizers; and, second, we identified factors that determine the use – or non-use – of P-based fertilizers. Although over 80% of farmers surveyed were aware of the value of fertilizers as a yield-increasing factor and were able to identify crops suffering from nutrient deficiency, only 10% used P-based fertilizers like single super phosphate (SSP) and another 11% used combinations of nitrogen, phosphorus, and potassium compound fertilizers and SSP for cowpeas. Reasons for not using P-containing fertilizers included high cost, poor availability in rural markets, and lack of awareness on the need to use P fertilizers. Additionally, many growers believed that cowpeas do not require fertilizers, especially if the previous crop had been maize. Our findings are important for strategies to increase the productivity of cowpeas among smallholder growers especially in the northern regions of Nigeria and beyond, where subsistence farming systems are prevalent. Increased cowpea production through the adequate use of inputs like P fertilizers will support Nigeria's effort to reduce its large imports of cowpea grain from neighboring countries. Our survey further demonstrated that P-containing fertilizers are crucial production inputs for increased cowpea production in these regions and in areas with similar traditional farming practices. Our results will benefit breeders, development partners, extension personnel, and other stakeholders in cowpea value chains.

## 1 Introduction

Cowpea (*Vigna unguiculata* L. Walp) is a popular leguminous crop in Nigeria and other countries in sub-Saharan Africa and provides food for over two hundred million people (AATF, 2010). Nigeria is the world's largest producer of grain cowpea, producing over 3.4 million metric tons of grains annually, which accounts for over 45% of global production (FAOSTAT, 2016). Owing to its large population (over 200 million people), Nigeria is also the largest consumer of grain cowpea, which is why substantial quantities of cowpea are imported into Nigeria from the neighboring countries, especially from the Republic of Niger, Chad, and Cameroon (Gómez, 2004; Langyintuo et al., 2003). This crop supplies a substantial amount of the daily protein needs of most people in major areas of consumption like Nigeria (Boukar et al., 2018; Singh et al., 2003).

The yield of cowpeas grown by local farmers is low – less than 600 kg ha^−1^ – compared to a potential of 1500–2500 kg ha^−1^ (AATF, 2012; ICRISAT, 2017a). This low yield can be attributed to biotic factors (e.g. insect pests, diseases, and parasitic weeds) as well as abiotic constraints which include low soil fertility, drought, and heat (ICRISAT, 2017a). Low soil fertility can be ascribed to deficiencies in nitrogen (N) and phosphorus (Bationo et al., 2002). Phosphorus (P) is a crucial macronutrient required by cowpea for optimum growth and development. It plays an important role in early root formation, crop quality, enhanced disease tolerance, seed formation, and several biochemical processes such as photosynthesis, respiration, energy storage and transfer, cell division, and enlargement (Johnston and Syers, 2009; Maharajan et al., 2017). Although cowpeas can fix a considerable amount of N in the presence of adequate P, the crop has difficulty accessing P contained in soil solution (Hussain, 2017; Krasilnikoff et al., 2003). Unfortunately, soils of most cowpea growing areas in West Africa—where soils are mostly acidic (low pH values), sandy and are deficient in extractable P available for plant use (Gyan-Ansah et al., 2016; Hussain, 2017). Sandy soils are generally poor, with low organic matter content, and deficient in major nutrients like N and P (Saidou et al., 2012; Sanginga et al., 2000).

Published research has established that, for soils very low in P and soils with P-fixing properties, the use of fertilizers formulated with P is recommended as a quick and easy fix (Kolawole et al., 2008; Bationo et al., 2002; Saidou et al., 2012). However, for several reasons that option has not been widely adopted by most smallholder cowpea growers. Most farmers in poor nations are unaware that P is a yield-boosting factor that needs to be applied to their legume fields (Horn et al., 2014). This non-use of synthetic P fertilizer by many smallholder farmers is compounded by the high cost of fertilizers (Bationo et al., 2002) and their unavailability in rural markets, which are places where farmers could easily access them if they were available (Olufowote and Barnes-Mcconnell, 2002).

## 2 Literature review

The Nigerian savannah soils are inherently low in N and P due to their low organic matter content and cation exchange capacity (Uyovbisere and Lombim, 1991) and their productivity is quickly decreasing due to continuous cultivation. Earlier works on fertilizer use in northern parts of Nigeria have focused on soil amendment practices with farmyard manure (FYM) to increase crop productivity (Bationo et al., 2002; Uyovbisere and Lombim, 1991). Though the use of FYM led to an increase in yield of major cereal crops, the nutrient supplied by FYM has been inadequate in sustaining crop productivity. The use of FYM at high rates on legumes like cowpea has led to a decrease in yield due to impaired fixation of atmospheric nitrogen by the crop, thereby making FYM on legumes as a main nutrient source without inorganic fertilizers unsustainable (Uyovbisere and Lombim, 1991). In addition, inadequate supply and handling cost of FYM has hindered the widespread use of FYM. Later fertilizer research in the savannah regions of Nigeria especially on groundnut and major cereals like maize, and sorghum were focused on the two most limiting soil nutrients; N and P (Goldsworthy, 1967; Lombin et al., 1985). After years of research on soils of Nigerian guinea Savanah regions, the potential of the soil resources are becoming evident given the right level of management practices and the key role played by inorganic fertilizers in maintaining soil fertility and crop yield has become more apparent especially as the traditional systems such as FYM, shifting cultivation and crop rotation are not able to deliver the required soil nutrients for improved productivity. The use of fertilizers is, therefore, a prerequisite to supporting higher levels of yields to feed the growing population (Uyovbisere and Lombim, 1991).

Current statistics showed the use of fertilizer especially N, P and K fertilizers in Nigeria has increased over what was obtained in the late 1980s, indicating increasing awareness in the use of fertilizers (FanWay, 2015; FEPSAN, 2014; Liverpool-Tasie et al., 2010). Notwithstanding this trend, fertilizer use on a hectare basis has remained low when compared with the world average per hectare. The use of P fertilizers on cowpea is lower in Nigeria especially in the major cowpea producing areas (Magani and Kuchinda, 2009; Saidou et al., 2012). P is the main limiting fertility for cowpea and its deficiency is widespread in most soils in the northern savannah areas due to acidic, and highly-weathered nature of the soils in these areas (Saidou et al., 2007; Uyovbisere and Lombim, 1991). Thus, P fertilizer is vital to achieving high yield in cowpea fields as its application facilitates optimum growth of the root and shoot systems, the formation of nodules and efficient biological nitrogen fixation for the crop. The element is also known to enhance the early maturity and good pod formation. Despite this importance associated with P fertilizers on cowpea, most farmers do not use the recommended type and rates of fertilizers for improved cowpea productivity partly due to poor awareness, inadequate supply and high cost of the fertilizers (Horn et al., 2014; ICRISAT, 2017a; Magani and Kuchinda, 2009).

## 3 Theoretical framework and objectives of the study

The present study is anchored on the theories concerning the dissemination and adoption of new technologies (Rogers, 2013). Rogers (2013) theory on diffusion of innovations posits that the rate of adoption or use of a new idea or innovation depends on the attributes of the new idea or practices such as the relative advantage of new technology, compatibility with existing values and practices, trialability, and observable results. An innovation in this context refers to an idea, or a practice that is perceived as new by an individual or other units of adoption regardless of actual newness of the concept, meaning here a technology may not be new in its entirety because even an old practice could be redesigned and reintroduced as a new practice (Rogers, 2013).

The use of fertilizers on crop plants is a well-known common practice among farmers that has been promoted and adopted in many farming systems (Agwu, 2004; Bationo et al., 2002; FanWay, 2015; Uyovbisere and Lombim, 1991). A synthesis of these characteristics of adopting new technology or improved practice reveals that for farmers to use a new practice, there are attributes that relate to the farmer and also to the improved practice and method of its dissemination that determines farmers' decision (Rogers and Shoemaker, 1971). The rate at which farming population uses or adopts a new agricultural technology or practice would depend on farmers’ circumstances, attributes of the technology itself, community norms and values, accessibility of the new technology, its relative affordability and the speed with which the technology is disseminated to the farmers (Purcell and Anderson, 1997; Rogers and Shoemaker, 1971). Furthermore, it has been observed that farmers are willing to use new technologies and adjust their investment when they are sure the new practice is good for them and assists in achieving their goals better (Purcell and Anderson, 1997).

It is well established that increased crop growth and productivity are associated with using the recommended fertilizers. For legumes like cowpea, there is a special need for P fertilizer to achieve higher production. Despite the obvious need for extra P, for high cowpea yield, and for an active P-fertilizer market in major cowpea production areas in Nigeria, this topic has received little attention. The knowledge and perception of farmers about the use of P and other inorganic fertilizers in cowpea fields have not been well investigated in major growing areas in Nigeria. This study's underlying hypothesis was that farmers believe that P and other fertilizers are not required for cowpea cultivation. Therefore, this research was designed and conducted, first, to assess the knowledge of farmers regarding their use of P fertilization in cowpea cultivation and, second, to identify the factors that determine farmers' use or non-use of P as a fertilizer for cowpea fields.

## 4 Materials and methods

### 4.1 Description of study areas

The study was carried out across 36 villages in 12 local government areas (LGAs) within three states in the Northern Guinea Savannah zone of Nigeria, one of the three agro-vegetational regions of the Nigerian savannah zones. Significant cowpea production has been reported previously for Kano, Kaduna, and Katsina states (Kormawa et al., 2002; Langyintuo et al., 2003; USAID, 2015).

At each survey site, all interviews were conducted in person and in the Hausa language. The design of this study was reviewed and approved by the Legumes and Oilseeds Research Review Committee of the Institute for Agricultural Research (IAR) Zaria as an internal interview process, this was in lieu of the institutional ethics committee, as an institutional ethics committee was not established at IAR Zaria when this work was conducted. In addition, the informed consent of interviewees was always obtained verbally before conducting the interviews and focus group discussion, as well as the option to participate or withdraw from the conduct of the study, was orally given to the participants.

Kano was the first state surveyed. It is the second-most densely populated state in Nigeria after Lagos, with more than 11 million inhabitants (NBS, 2012). It lies mostly in the Sudan savannah zone, with pockets of other types of savannah ecologies; these provide a wide range of growing periods with an annual rainfall of 500–1000 mm. The four LGAs surveyed in Kano were Albasu, Bunkure, Tsanyawa, and Minjibir. Cowpea is grown by most farmers in Kano and serves as an important source of income and food (Kormawa et al., 2002; Langyintuo et al., 2003). The state is home to *Dawanau* market, the biggest international cowpea grain market in West Africa, which serves as the main import-export terminal for cowpea grain (Gómez, 2004). The second state surveyed was Kaduna, which is in the central part of northern Nigeria and has a population of over 7 million (NBS, 2012). It is one of the main cowpea producing areas in Nigeria, with an annual rainfall of 1000–1500 mm (NAERLS et al., 2017). The four LGAs surveyed were Birnin-Gwari, Giwa, Kajuru, and Makarfi. Katsina was the third state surveyed, located in the north-western part of Nigeria, with a population of over 6 million. It is also a major cowpea-producing zone and has three main ecological zones: Sudan, Sahel, and Northern Guinea Savannah. The four LGAs surveyed there were Matazu, Kaita, Danja, and Dandume. The annual rainfall of this state is 1000–1600 mm (NAERLS et al., 2017).

### 4.2 Sampling procedure

A two-step sampling procedure was undertaken to select cowpea farmers for the study. The first step was to identify the major production areas in Nigeria based on published literature (Kormawa et al., 2002; USAID, 2015) and randomly select three states in the northern ‘cowpea belt,’ a term used to describe major cowpea production zones (Ishiyaku and Aliyu, 2013). The second step was the random selection of 420 households involved in cowpea cultivation across the selected study sites with the help of village extension agents from the various areas. An average of 10 farmers per village were selected from each of the LGAs per state using a stratified random sampling procedure. Farmers were selected if they had grown cowpea in the previous season.

### 4.3 Questionnaire design and data collection

Semi-structured questionnaires, developed and pre-tested by the research team, were used to gather information from farmers. The validity of the instrument was tested by a panel of expert scholars in social sciences and measurement techniques at the Institute for Agricultural Research (IAR) and Ahmadu Bello University (ABU), Zaria. The panel of scholars recommended certain amendments in the wordings, number of items and arrangement of the scale. Those recommendations were effected in the instrument before data collection to the satisfaction of the measurement scholars. A pilot study was conducted to test the reliability of the Likert-type scales in the instrument. Reliability was established as the instrument recorded Cronbach's alpha value of 0.87, which was well above the acceptable threshold of 0.7 (Zanolin et al., 2007; Field, 2009; Pallant, 2011). The questionnaires were used to collect data on socio-economic characteristics, the experience of cowpea production, fertilizer use, and knowledge of P-based fertilizers in cowpea fields. Additionally, the knowledge of farmers about nodules found on the cowpea root system was investigated because nodules are the key element responsible for the N-fixation ability of legumes, including cowpeas. Data were collected from a total of 420 farmers in these villages (an average of 35 farmers from four villages in each LGA, i.e. 35 farmers × 4 LGAs × 3 states). The survey was administered by trained enumerators and the data collected were validated with information obtained via focus group discussion sessions with key interviewees, and with personal observation during the survey.

A five-point Likert scale with seven questions was used to further understand the thinking of farmers concerning the use of P-based fertilizers on cowpea fields. Responses were coded on a five-point Likert scale: 1 = strongly disagree, 2 = disagree, 3 = undecided, 4 = agree, and 5 = strongly agree (Allen and Seaman, 2007). Factor analysis was used to help determine important variables influencing farmers’ perceptions of P-based fertilizers. Since it is important to test the adequacy of sampled respondents for factor analysis to be valid, the Kaiser–Meyer–Olkin measure of sampling adequacy (KMO-MSA) was estimated based on the work of Nkansah (2011).

A binary logit model was used to study the farmers’ choice of using or not using synthetic P fertilizers on cowpea. These options were treated as two mutually exclusive events, such that when a farmer chose the option of using synthetic P fertilizers, the other option by default was not chosen (Horn et al., 2014). The use of P-based fertilizer for cowpea was modeled as a dependent variable with a binary choice: the value was 1, if the farmer used P-based fertilizer in cowpea fields; otherwise, the value was 0. The logit model is appropriate for responses with two possible outcomes such as 1 or 0, yes or no, or present or absent (Naziri et al., 2014; Mendesil et al., 2016). The use and non-use of P-based fertilizers by cowpea farmers served as the dependent variable while the independent (explanatory) variables were sex, marital status, age, household size, level of formal education, experience in growing cowpea, ability to detect nutrient deficiency symptoms, cropping systems, attendance of field-days, and contacts with extension agents (Table 1).

**Table 1:** List and description of variables used in the binary logit model.

Variable	Description	Variable type	Units
**Dependent variable**			
Use P-fertilizer	Farmer's use of P on cowpea	Dummy	1 = yes, 0 = no
**Independent variable**			
Sex	Farmer's sex	Dummy	1 = male, 0 = female
Status	Farmer's marital status	Dummy	1 = married, 0 = single
Age	Farmer's age	Years	Continuous
Household	Size of farmer's household	Persons	Continuous
Education	Farmer's formal education	Levels	Continuous
Experience	Experience in cowpea cultivation	Years	Continuous
Def-Symptoms	Ability to identify nutrient deficiency symptoms	Dummy	1 = yes, 0 = no
Cropping	Cowpea cultivation system	Dummy	1 = sole, 0 = mixed
Field-day	Attendance of cowpea field-days	Dummy	1 = yes, 0 = no
Extension-Contact	Access to cowpea production information from extension agents	Dummy	1 = yes, 0 = no

### 4.4 Focus group discussions

Focus group discussions (FGDs) were conducted during January–March 2017. A group of 8–10 participants per site was interviewed to gain a detailed insight into some of the questions asked in the questionnaire. The criterion for inclusion in the FGD was for a farmer to have grown cowpea in the previous season. The groups in Kaduna and Katsina included male and female growers whereas, in Kano, separate groups of men and women were formed to conform to cultural norms. Village extension agents from the Agricultural Development Programme of the target states organized and facilitated the discussion sessions. The FGD sessions were led by the principal researcher and, in some areas by other facilitators, who usually introduced the topics for discussion and guided members of the group toward effective participation.

### 4.5 Data analysis

Data collected from questionnaires and FGD sessions were coded, entered into spreadsheets, and analyzed using SPSS 20.0, and NLOGIT software version 4.0 was used for the analysis of factors determining the use and non-use of P fertilizers. Results were summarized and presented using descriptive statistics, factor analysis and logit regression. As an exploratory study aimed at spurring interest and further investigations, this paper leaned heavily on descriptive statistics to report the knowledge of farmers regarding the use of phosphorus (P) fertilizer in cowpea cultivation. The main aim of the logit analysis was to describe the way in which the use of P-containing fertilizers was influenced by the explanatory variables stated in Table 1. Empirically, the model for estimating the determinants of the probability of farmers using P-containing fertilizer is as follows (Verbeek, 2004):

$$\ln\left[\frac{P_{x}}{1-P_{x}}\right]=\;\beta_{o}+\;\sum \beta i\;Xi$$

where *P*_*x*_ is the probability of an event occurring (value of 1 if the farmer uses P-containing fertilizer; otherwise, the value is 0), *β*_*o*_ is a constant term, and *βi* is a coefficient associated with the independent variable *Xi*. Several independent variables likely to influence the choice of farmers using synthetic P-based fertilizers on cowpea were identified and used in the model (Table 1).

## 5 Results

### 5.1 Socio-economic metrics of the respondents

The majority (85%) of cowpea farmers (n = 420) across the three states of the study were male (Table 2). This is probably due to the religious and cultural background of the respondents, since most women do not engage in direct crop-cultivation activities such as land preparation, planting, weeding, and field management. Women are mostly responsible for post-harvest processing, threshing, and winnowing. However, we noted that in 40% of the LGAs surveyed, there was a good representation of female cowpea producers, namely in Birnin-Gwari, Bunkure, Kajuru, Makarfi, and Tsanyawa (Table 2). In these areas, female cowpea farmers had good contact with extension agents and were involved in cooperative activities. At Tsanyawa, most of the women farmers had post-secondary education and used hired labor for the management of their farms.

**Table 2:** Gender distribution of respondents.

Local Government Area	Gender of respondent			Percentage by gender	
	Male	Female	Total respondents	Male	Female
Albasu	35	3	38	92	8
Birnin-Gwari	30	5	35	86	14
Bunkure	25	10	35	71	29
Dandume	29	0	29	100	0
Danja	36	0	36	100	0
Giwa	37	5	42	88	12
Kaita	30	0	30	100	0
Kajuru	24	17	41	59	41
Makarfi	19	11	30	63	37
Matazu	32	3	35	91	9
Minjibir	35	0	35	100	0
Tsanyawa	24	10	34	71	29
Total respondents	356	64	420	85	15

Most of the farmers were married (96%), indicating that the establishment of a family unit is an important cultural norm in these farming communities (Table 3). The age of farmers cultivating cowpea varied widely: most farmers were 30–50 years old across all sites. Our surveys showed that the level of formal education among the respondents was generally low: over 37% had no level of formal education (Table 3). Approximately 21% of the respondents had received some formal education including at least six years of primary and secondary education, and post-secondary qualification (mostly two-year certificate courses) in areas close to urban centers like Albasu, Dandume, Matazu, and Tsanyawa. Most farmers (37%) in some areas had informal Qur'anic education and were able to read and write in the local language (Hausa) using Arabic alphabets, a system known as “Ajami” (Dobronravine and Philips, 2004).

**Table 3:** Socio-economic attributes of 420 cowpea farmers surveyed across study areas.

Attributes of the respondents		Frequency	Percent
Marital status	Married	402	95.7
	Single	18	4.3
Age	11–20	9	2.1
	21–30	56	13.3
	31–40	127	30.2
	41–50	108	25.7
	51–60	91	21.7
	>60	29	6.9
Level of education	Primary	88	21.0
	Secondary	89	21.2
	Tertiary	88	21.0
	Informal	155	36.9
Years of cowpea production	1–10	211	50.2
	11–20	113	26.9
	21–30	66	15.7
	31–40	23	5.5
	>40 years	7	1.7

Half the cowpea farmers interviewed (50%) had more than 10 years of experience in cowpea cultivation, 27% had up to 20 years of experience, while approximately 2% had been growing cowpeas for over 40 years. The range of experience in cowpea cultivation implies that these farmers should be aware of the traditional cowpea production practices such as planting a large-seeded landrace, intercropping with staple cereals, and late-season planting of the cowpea crop (Table 3).

### 5.2 Cowpea farmers’ knowledge and use of P-based fertilizers

**Figure 1:**
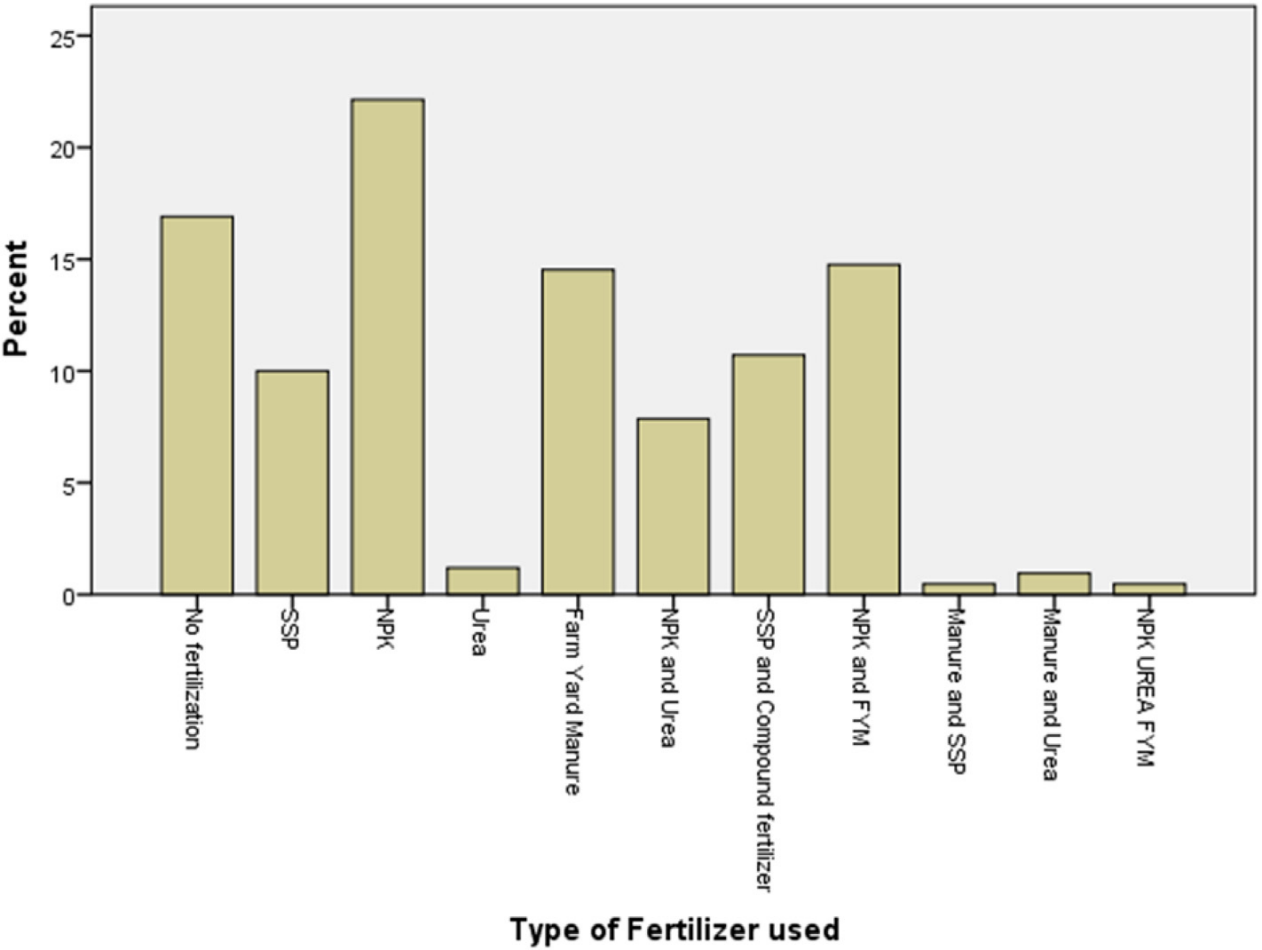
Percentage of cowpea farmers (n = 420) using different types of fertilizers, where SSP is single super phosphate, NPK as nitrogen–phosphorus–potassium, and FYM is farm-yard manure.

Most farmers used some form of fertilizer. This indicates that farmers were aware of the importance of fertilizers in crop productivity. Several kinds and combinations of fertilizers were used, including nitrogen–phosphorus–potassium (NPK), single super phosphate (SSP), urea, farm yard manure (FYM), NPK + urea, SSP + NPK, NPK + FYM, FYM + SSP, FYM + urea, and NPK + urea + FYM (Figure 1). FYM is a combination of animal droppings (such as poultry droppings), cow dung, ashes, and refuse. Most farmers did not use P-containing fertilizers to grow cowpea. Only 10% used SSP, a P-based commercial fertilizer, while 11% used a combination of SSP + NPK, 0.5% used FYM + SSP, and 17% added no fertilizers. During the interviews, many farmers expressed the belief that cowpea does not require fertilizers. This belief was due to their traditional practice of not applying fertilizers to cowpea fields; hence, they did not systematically add the recommended fertilizers to their fields.

Farmers’ knowledge and awareness of the benefit of P to cowpea cultivation and problems associated with its deficiency were further assessed on a five-point Likert-type scale with three (3) as mean. Summated scales are known to enhance reliability through multivariate measurement (Hair et al. 2010). Farmers that scored 3 and above on an item were considered knowledgeable (yes) on such component. Any score of less than 3 was considered otherwise (no). Over 81% could identify plants suffering from inadequate nutrients in the soil (Table 4). Most farmers (88.2%) had noticed the presence of nodules on cowpea roots (Table 4). This was indicated during the surveys with photographs of cowpea roots with nodules attached. Nitrogen-fixing nodules are an important characteristic of legumes, enabling the biological fixation of atmospheric nitrogen for plant use. However, although most farmers had noted the presence of root nodules, 78% were unaware of the role these nodules play. The results of this survey are shown in Table 4. Furthermore, from the focus group discussion (FGD), a small proportion of farmers perceived nodules to be harmful to the health of the plant (as is the case with attachments of *Striga*, a parasitic plant).

**Table 4:** Farmers’ recognition of nutrient deficiency in cowpea, and of the presence and function of root nodules.

Farmer responses	Number of respondents	Percent
**Deficiency recognition**		
Yes	337	80.2
No	83	19.8
**Knowledge of nodules**		
Yes	370	88.2
No	50	11.8
**Knowledge of the role of root nodules in cowpea**		
Yes	94	22
No	326	78

### 5.3 Assessment of farmers’ perceptions of phosphorus fertilization for cowpea fields

Descriptive statistics of the Likert items showed that the Likert item “phosphorus increases cowpea yield” accounted for most of the variation with the highest mean of 4.37. The KMO-MSA test yielded a value of 0.620 (Table 5), which falls within the range of acceptable values for a satisfactory factor analysis to be undertaken (Kaiser and Rice, 1974). Bartlett's test of sphericity was highly significant (Table 5), so the null hypothesis was rejected. Several Likert items were influential in determining farmers' perception of the use of P-based fertilizers (Table 5). The first three components were retained for further interpretation, since they explained over half of the variation (61.1%) in the dataset (Table 6).

**Table 5:** Descriptive statistics on Likert items for how cowpea farmers perceive P fertilization, and a sampling adequacy test.

Variables (Perception)	Mean	Standard Deviation
P reduces growth and vigor	1.94	1.249
P increases cowpea yield	4.37	1.008
P use increases cost of production	2.97	2.908
P use is labor intensive and time-consuming	2.68	1.332
I do not use P because of no prior knowledge about its use	2.94	1.469
I do not use P because it is expensive	2.80	1.429
I do not use P because it is unavailable in the market	2.90	1.471
**Sampling adequacy and strength of relationship among variables**		
Kaiser–Meyer–Olkin Measure of Sampling Adequacy	0.620	
Bartlett's Test of Sphericity (Approximate Chi-Square)	268.093	
Degrees of Freedom	21	
Significance	0.000

**Table 6:** Variables and their contribution to total variance of the components.

Variables (Factors)	Components		
	1	2	3
P reduces growth and vigor	NA	0.790	NA
P increases cowpea yield	NA	−0.799	NA
P use increases the cost of production	0.713	NA	NA
P use is labor intensive and time-consuming	0.648	NA	NA
I do not use P because of no prior knowledge about its use	NA	NA	NA
I do not use P because it is expensive	0.775	NA	NA
I do not use P because it is unavailable in the market	NA	NA	0.869
Percent variance explained by components	24.6	21.1	15.2
Percent cumulative variance explained by components	24.6	45.9	61.1

NA = no contribution to the component by the variable.

### 5.4 Determinants for use and non-use of phosphorus-based fertilizers among cowpea farmers

The binary logit model used to model factors influencing the use and non-use of P fertilizers among cowpea farmers showed three independent variables that influenced positive and significant prediction of P use on cowpea: knowledge of nutrient deficiency symptoms, attendance of field-days, and contacts with extension agents (Table 7).

**Table 7:** Binary logit outputs on variables influencing the use of P fertilizers.

Variable (see Table 1)	Coefficient	SE	b/St. Er.	P[|Z|>z]	Mean
Constant	2.895∗∗	1.041	2.772	0.007	NA
Sex	−0.741	0.312	−2.374	0.018	1.15
Status	−0.370	0.539	−0.686	0.493	1.04
Age	0.066	0.118	0.557	0.577	4.72
Household-size	0.006	0.014	0.408	0.683	13.49
Education	−0.162	0.982	−1.649	0.099	2.74
Experience-cowpea	−0.282	0.133	0.212	0.832	1.81
Def-Symptoms	−0.986∗∗	0.274	−3.598	0.0003	1.19
Cropping-system	−0.105	0.107	−0.977	0.329	2.28
Field-days	0.453∗	0.206	2.193	0.028	0.50
Extension-Contacts	0.380∗	0.164	2.324	0.020	0.78

∗P < 0.05; ∗∗P < 0.01; N = 420; log likelihood = −242.6675; LR x^2^ (11) = 53.39966; Prob > x^2^ = 0.0000; McFadden pseudo-R^2^ = 0.0991205; NA = not applicable, SE = standard error, and P is probability of Z-score.

### 5.5 Access of farmers to cowpea field-days and to agricultural extension services

Over half the farmers (60%) reported no attendance at field-days on cowpea cultivation and only about 10% attended more than five field-days. The trend was similar for contacts with agricultural extension agents: 44.3% had no contact, 33.1% had one to five visits, and only 23% had more than five visits to or by extension personnel for education in cowpea production practices (Table 8). This clearly demonstrates the need to provide farmers with training and education on cowpea practices to achieve a sustainable cowpea food system. Some of the farmers that reported attendance of field-days were those that had participated in the recent USAID-sponsored upscaling projects at Matazu, Minjibir, and Albasu, while others were farmers collaborating with the International Institute of Tropical Agriculture (IITA) in the out-growers program at the Giwa LGA.

**Table 8:** Percentage of farmers attending field-days and having contact with extension agents.

No. of times	Attendance of field-days		Contact with extension agents	
	Frequency	Percentage	Frequency	Percentage
None	251	59.8	186	44.3
1–5	129	30.7	139	33.1
>5	40	9.5	95	22.6
Total	420	100	420	100

## 6 Discussion

The study was undertaken to determine the extent to which P fertilizers are used by cowpea farmers and to understand the level of knowledge that farmers have regarding the use of fertilizers in their cowpea fields. The KMO-MSA value of 0.620 in this study was above the accepted minimum value (0.50), implying that the respondent sample size was adequate for the study. Several reports had established that the number of respondents adequate for a satisfactory factor analysis should be 300 and above (Field, 2009, p. 647). Therefore, our sample size of 420 used for investigating the research questions met this requirement.

Previous research has shown that the majority of cowpea growers in the northern parts of Nigeria are men, in contrast to the practice in some Southern and West African countries like Zambia and Burkina Faso where women are the dominant cowpea producers (Gómez, 2004; Nkongolo et al., 2009). The results of our study corroborate the findings in northern Ghana (Akpalu et al., 2014) and north-eastern Nigeria (Iya and Kwaghe, 2007) that more men than women were involved in cowpea farming and that women were involved more in post-harvest operations like threshing, cleaning, and winnowing grains. Women in these areas were mostly of the Muslim faith and practice purdah (the requirement that women cover their bodies and avoid mingling with men other than their husband or close relatives) (Yusuf, 2014; Zakaria, 2001). Previous reports have also indicated that most heads of households in the northern parts of Nigeria prefer that their wives and daughters do not participate in farm operations (Rahman, 2008; Umar et al., 2014).

We also found that most of our respondents had no formal education, thus corroborating the findings of Tijjani et al. (2015) that most cowpea farmers in Katsina state, one of the states surveyed in this study, had no formal education. A USAID-sponsored study in the two northern Nigerian states of Sokoto and Bauchi explored the reasons for the low level of formal education among poor households in northern Nigeria. They found that access to formal education (primary, secondary, and post-secondary qualifications) is low in the northern parts of Nigeria—below the national average (USAID, 2013). Similar reports have also indicated that reading skills are dismal among third-grade pupils, and that most children in this region attend informal Qur'anic and Islamic schools with no focus on basic English reading and skills in other subjects (Hatch, 2012; USAID, 2013). Low formal education among cowpea farmers has been reported elsewhere in northern Ghana, where over half (57%) of cowpea farmers in that region had no formal education (Akpalu et al., 2014). The poor participation in the formal education system could be among the main factors limiting the use of improved technologies among these farmers. The education of farmers is key to the success of any agricultural development program; thus, there is an urgent need to educate smallholder farmers about new farming technologies such as the use of P fertilizers and other improved practices.

The findings from this study revealed that, cowpea farmers were aware of the important role that fertilizers play in having healthy plants in the field, but most did not use any P-containing fertilizer formulations for cowpeas. Earlier reports have shown fertilizer use in Nigeria to be low (13 kg/ha), far below the rate of 200 kg/ha recommended by the United Nations Food and Agriculture Organization (Liverpool-Tasie et al., 2010). Reasons provided by farmers for not using P-based fertilizers on cowpea plants included the lack of awareness regarding the need to use P fertilizers like SSP or other P-based fertilizers for cowpea cultivation and the poor availability of these products in rural markets. Where these products were available, farmers mentioned the high cost associated with them. These agree with some of the findings of Liverpool-Tasie et al. (2010), who indicated that low use of fertilizers in Nigeria was due to the high cost of the products and their limited availability in urban and rural markets. In addition, FanWay (2015) pointed out that Nigerian farmers were constrained by inadequate technical knowledge, research, and dissemination of new findings. Similarly, it has been noted elsewhere that most farmers in developing countries do not have sufficient access to phosphate fertilizers in their communities (Magani and Kuchinda, 2009; Chimungu and Lynch, 2014); similar findings were reported among cowpea growers in Namibia (Horn et al., 2014). Moreover, many cowpea farmers across the areas surveyed in this study believed that their cowpea fields did not require fertilizers and hence did not systematically apply the required amount of fertilizers recommended by the research institutions. Similar findings were reported among cowpea growers in northern Namibia (Horn et al., 2014).

Most of Nigeria's arable land suffers from poor soil fertility having low organic matter content, and severe N deficiency in more than 80% of the country's land (N is below 0.1%), over 75% of the Nigerian arable land had P deficiency (P is less than 10 mg P/kg of soil) and greater than 60% of the arable land is in severe potassium (K) deficiency (K is less than 25 mg K/kg of soil) (FanWay, 2015). This poor soil fertility is mainly due to the low use of fertilizers and to other practices such as bush burning and the removal of crop residues after harvest for animal feed and for use in the construction of local structures (Bationo et al., 2002). The low usage of fertilizers in the region is primarily due to the high cost of fertilizers, especially in countries like Nigeria, where most of the fertilizers and their raw materials are imported (Banful et al., 2010; FanWay, 2015). Another reason is untimely fertilizer availability in areas where farmers could have easy access to them (Banful et al., 2010). These factors underscore the need to create and sustain platforms to continuously educate and guide farmers on the use of recommended fertilizer packages for growing cowpea.

The results of the binary logit analysis revealed that the use of P-based fertilizer was strongly associated with the ability of farmers to determine nutrient deficiency, their attendance of field-days, and their contact with agricultural extension personnel. This is consistent with the opinion that the education of farmers is important for the successful adoption of farming technologies and recommendations (Godfray et al., 2010; Mendesil et al., 2016).

This model (binary logit) was used to understand factors determining the use of P fertilizers for cowpea. Similarly, binary models have been used previously to estimate factors influencing knowledge of Napier stunt disease (Khan et al., 2014), farmers’ knowledge of pea weevils (Mendesil et al., 2016), and the decision to use pesticides in vegetable crops (Sharma et al., 2015). Additionally, from interviews and focus group discussion sessions, we established that the use of a P-based fertilizer like SSP for cowpea production was common only among growers with some formal education and those who had contact with projects such as those run by the IITA, and with USAID-sponsored cowpea upscaling projects in the northern Nigeria states of Kano, Katsina, and Sokoto (Adetonah et al., 2016; ICRISAT, 2017b).

Cowpea farmers in this study had poor exposure to guidance on the production of the crop and did not have enough contact with extension agents. This is probably due to the low farmer-to-extension agent ratio (1:10,000) in Nigeria (Haruna and Abdullahi, 2013; NAN, 2016). This is in contrast to the World Bank recommendation of 1:800–1000 extension agent-to-farm households’ ratio. Extension agents are important to increase P-fertilizer use because they can help educate farmers on the need for P-fertilizer use on cowpea, thereby leading to increased P use and increased cowpea productivity. However, the extension agents in Nigeria are over-stretched and only a few farmers can access and benefit from their extension services (Banful et al., 2010). It has been shown that most extension services in the rural areas of Nigeria put more emphasis on the transmission of improved seeds and rarely transmit information on other technologies like fertilizer use and management practices that are important to achieve increased productivity (Banful et al., 2010). Banful et al. (2010) further demonstrated that most village extension agents have limited knowledge of appropriate fertilizer types and their recommended rates for different crop plants. They also pointed out that poor access to farm inputs such as fertilizers is a serious constraint for both male and female farmers, but they considered that low fertilizer use among farmers was due to lack of availability and not high cost of the products (Banful et al., 2010). Contact with extension personnel provides opportunities for agricultural information exchange, including better crop management practices and soil fertility improvement strategies. Such information exchange results in providing farmers with better knowledge about factors that can increase their yields. Information guides on the use of agricultural inputs might be an important way to educate smallholder farmers on knowledge about crop management practices (Belt et al., 2015).

## 7 Conclusions

Cowpea growers in this study did not use recommended fertilizer types and rates for cowpea; they were thus inadvertently contributing to the low yield that characterizes African agriculture for most crops. Most of the 420 farmers that participated in the study were aware that fertilizers are important for crop growth and healthy development, but they did not know the appropriate fertilizer recommendations for cowpeas. Those who were aware of the need to use P fertilizers on cowpea complained of the high purchase price, putting this forward as the reason for not using these fertilizers. The use and non-use of P-based fertilizers was also influenced by the farmers’ knowledge of nutrient deficiency symptoms, exposure to training and guidance from field-day events, and interaction with extension personnel.

On the basis of information obtained from the key informants during the surveys and FGDs, many of the farmers are willing to use P-based fertilizers like SSP if these are given to them or made available in the rural markets at subsidized prices. Our work shows that it is important to consider farmers’ knowledge and perceptions when designing new agronomic approaches, as this will greatly facilitate the diffusion and adoption of new and improved technologies among farmers. Our findings will inform the decision-making process and planning of programs aimed at improving sustainable cowpea food systems.

## Declarations

### Author contribution statement

Mohammed B. Saba: Conceived and designed the experiments; Performed the experiments; Analyzed and interpreted the data, Wrote the paper.

Muhammad F. Ishiyaku, Pangirayi B. Tongoona, Vernon Gracen, Daniel K. Dzidzienyo, Muhammad L. Umar, Sulaiman Umar: Conceived and designed the experiments; Analyzed and interpreted the data, Wrote the paper.

### Funding statement

This is work was supported by the 10.13039/100000865Bill and Melinda Gates Foundation [OPP1131397] through the PEARL II grant to the Ahmadu Bello University, Zaria-Nigeria.

### Competing interest statement

The authors declare no conflict of interest.

### Additional information

No additional information is available for this paper.
